# Supplementary material for: Resveratrol intervention attenuates chylomicron secretion via repressing intestinal FXR-induced expression of scavenger receptor SR-B1
Source: Nat Commun. 2023 May 9;14:2656. doi: 10.1038/s41467-023-38259-1 (PMC10169763; doi:10.1038/s41467-023-38259-1)
Supplement: Supplementary file 1 — Supplementary Information [file 41467_2023_38259_MOESM1_ESM.pdf]

Supplementary Information

Table S1. Primer sequences utilized for real-time PCR.

Species	Genes	Forward sequence (5’-3’)	Reverse sequence (5’-3’)
	Cd36	TATTGGTGCAGTCCTGGCTG	CTGCTGTTCTTTGCCACGTC
	Slc27a4	TGAGATGGCCTCAGCTATCTG	TGCCCGATGTGTAGATGTAGAA
	Fabp2	GTGGAAAGTAGACCGGAACGA	CCATCCTGTGTGATTGTCAGTT
	Mttp	AGCCAGTGGGCATAGAAAATC	GGTCACTTTACAATCCCCAGAG
	Apoa4	TCAGAAGACGGATGTCACTCA	ATGCGGTCACGTAGGTCCT
	Apob	TTGGCAAACTGCATAGCATCC	TCAAATTGGGACTCTCCTTTAGC
	Fabp1	ATGAACTTCTCCGGCAAGTACC	CTGACACCCCCTTGATGTCC
	Scarb1	TGTACTGCCTAACATCTTGGTCC	ACTGTGCGGTTCATAAAAGCA
	Sar1b	TTCCTTGGATTGGATAATGCCG	GCCAGCAATAGTAAGCTCTTCTG
	Sec13	GAACACTGTGGACACCTCTCA	CTCCATTCCGCACATCGAAAA
	Vamp7	GACAACTTACGGTTCAAGAGCA	TCTCCACGTTGAGCAACTAAATC
mouse	*Acaca*	CACTCCTTAGAGAGGGGTCA	TAACTTCCCAGCAGACGGTG
	Fasn	AGAAGTGCAGCAAGTGTCC	GGTCGGATGAGGGCAATCTG
	Me1	AGGCTATTGTGGTAACTGATGG	CAGGAAGGCGTCATACTCAG
	Scd1	TGGAGCCACAGAACTTACAAG	GTTTTCCGCCCTTCTCTTTG
	Dgat1	TCCGTCCAGGGTGGTAGTG	TGAACAAAGAATCTTGCAGACGA
	Mogat2	TGGGAGCGCAGGTTACAGA	CAGGTGGCATACAGGACAGA
	Acadm	AGGGTTTAGTTTTGAGTTGACGG	CCCCGCTTTTGTCATATTCCG
	Acadl	TCTTTTCCTCGGAGCATGACA	GACCTCTCTACTCACTTCTCCAG
	Ehhadh	AGCTGTTTATGTACCTTCGGG	CTGCTTTGGGTCTGACTCTAC
	Acox1	AGAAGAGTGCCACCATGGTG	GCATGAAGAAACGCTCCTGC
	Cpt1α	TGCATACCAAAGTGGACCCC	GTGCTCTGCAAACATCCAGC
	Il1b	GAAATGCCACCTTTTGACAGTG	TGGATGCTCTCATCAGGACAG

mouse	*Il6*	CTGCAAGAGACTTCCATCCAG	AGTGGTATAGACAGGTCTGTTGG
	Tnf	CGTCAGCCGATTTGCTATCT	CGGACTCCGCAAAGTCTAAG
	Ifng	ATGAACGCTACACACTGCATC	CCATCCTTTTGCCAGTTCCTC
	Ccl2	TTAAAAACCTGGATCGGAACCAA	GCATTAGCTTCAGATTTACGGGT
	Ddit3	CTCGCTCTCCAGATTCCAGTC	CTTCATGCGTTGCTTCCCA
	Hspa5	ACTTGGGGACCACCTATTCCT	ATCGCCAATCAGACGCTCC
	Xbp1	GACAGAGAGTCAAACTAACGTGG	GTCCAGCAGGCAAGAAGGT
	Nr1h4	GCTTGATGTGCTACAAAAGCTG	CGTGGTGATGGTTGAATGTCC
	Nr0b2	TGGGTCCCAAGGAGTATGC	GCTCCAAGACTTCACACAGTG
	Fgf15	ATGGCGAGAAAGTGGAACGG	CTGACACAGACTGGGATTGCT
	Slc51a	ACACAGCAGAGCTTCTGGAA	TTGATGGGGCAAAGGGTGTT
	Slc51b	AGATGCGGCTCCTTGGAATTA	TGGCTGCTTCTTTCGATTTCTG
	Cyp7a1	GGGATTGCTGTGGTAGTGAGC	GGTATGGAATCAACCCGTTGTC
	Cyp7b1	GGAGCCACGACCCTAGATG	TGCCAAGATAAGGAAGCCAAC
	Cyp27a1	ACACGGATGCCTTAAACGAGG	GCAGCCAATCCTTTTCTCAAAC
	Cyp2c70	AGTATGGCCCTGTGTTTACTGT	GCCTTGGCTGGTTCTACTGAG
	Cyp8b1	CCTCTGGACAAGGGTTTTGTG	GCACCGTGAAGACATCCCC
	Actb	TCATGAAGTGTGACGTTGACA	CCTAGAAGCATTTGCGGTG
human	*SCARB1*	CCTATCCCCTTCTATCTCTCCG	GGATGTTGGGCATGACGATGT
	RELA	GTGGGGACTACGACCTGAATG	GGGGCACGATTGTCAAAGATG
	IL6	ACTCACCTCTTCAGAACGAATTG	CCATCTTTGGAAGGTTCAGGTTG
	TNF	CCTCTCTCTAATCAGCCCTCTG	GAGGACCTGGGAGTAGATGAG
	IL1B	ATGATGGCTTATTACAGTGGCAA	GTCGGAGATTCGTAGCTGGA
	CCL2	CAGCCAGATGCAATCAATGCC	TGGAATCCTGAACCCACTTCT
	NR1H4	AACCATACTCGCAATACAGCAA	ACAGCTCATCCCCTTTGATCC
	NR0B2	TGGCTTCAATGCTGTCTGGA	AATGGACTTGAGGGTGGAGG
	FGF19	GACTGTGCTTTCGAGGAGGA	GGGCATGGTCTCAGTTACTTCT
	ACTB	CATGTACGTTGCTATCCAGGC	CTCCTTAATGTCACGCACGAT

Table S2. Nucleotide sequences of primers for genotyping.

Gene	Forward sequence (5'-3')	Reverse sequence (5'-3')	Size (bps)
floxed SR-B1	GCACAGAGGACCCAACAG CGCACAAAATGG	GCTGGGATTCAAGGTGTG TGCCACCACCTAC	149/188[a]
Vil-cre 1000 control	GCCTTCTCCTCTAGGCTCGT	TATAGGGCAGAGCTGGAG GA	182
Vil-cre 1000 transgene	GCCTTCTCCTCTAGGCTCGT	AGGCAAATTTTGGTGTAC GG	150

[a]149 bps for WT allele and 188 bps for the floxed allele.

Table S3. Nucleotide sequence of primers utilized for ChIP in mouse *Scarb1* promoter.

Site	Forward sequence (5'-3')	Reverse sequence (5'-3')	Size (bps)
RelA motif	GGCTTAAAGGCTCTGGCTGT	GAGGGGCTTATCAGACTGCC	211
Intron2	AGGTGCGCTCTGTTTGGAAT	GCCCCTGAGCTCTGAAAACT	211

Table S4: Relative expression of genes listed in Figure 2E

Gene	LFD (fold)		HFD (fold)		HFR (fold)		*P* value (LFD VS. HFD)	*P* value (HFR VS. HFD)
	Mean	SD	Mean	SD	Mean	SD		
Cd36	0.71	0.33	1.05	0.32	1.17	0.52	0.038	0.319
Slc27a4	0.91	0.17	1.00	0.15	0.95	0.11	0.118	0.544
Fabp2	0.95	0.16	1.01	0.16	0.96	0.23	0.494	0.631
Mttp	0.87	0.28	1.01	0.17	0.98	0.24	0.140	0.957
Apoa4	0.62	0.30	1.04	0.31	0.85	0.30	0.003	0.380
Apob	0.94	0.28	1.16	0.37	1.05	0.22	0.077	0.442
Scarb1	0.21	0.14	1.11	0.50	0.74	0.44	0.000	0.026
Sar1b	0.84	0.26	1.03	0.27	0.89	0.23	0.071	0.308
Vamp7	0.98	0.16	1.02	0.19	0.93	0.21	0.687	0.475
Fabp1	0.73	0.40	1.02	0.20	1.09	0.37	0.034	0.531
Sec13	0.90	0.23	1.00	0.10	0.95	0.24	0.205	0.501
Fasn	1.30	0.37	1.02	0.09	1.46	0.66	0.202	0.053
Acaca	1.19	0.35	1.05	0.18	1.11	0.20	0.215	0.519
Scd1	1.11	0.49	1.08	0.37	0.99	0.60	0.836	0.810
Me1	0.59	0.22	1.08	0.53	1.40	0.83	0.080	0.323
Mogat2	0.99	0.28	0.99	0.18	1.20	0.24	0.871	0.126
Dgat1	0.75	0.24	0.99	0.21	0.94	0.17	0.017	0.437
Acadm	0.87	0.15	1.02	0.24	1.26	0.27	0.185	0.042
Acadl	0.86	0.13	1.02	0.23	1.31	0.43	0.283	0.059
Acox1	1.30	0.49	1.02	0.52	1.20	0.72	0.611	0.861
Ehhadh	0.80	0.22	1.01	0.37	1.48	0.52	0.190	0.047
Cpt1a	0.90	0.21	1.00	0.16	1.48	0.44	0.456	0.004

Table S5: Relative expression of genes listed in Figure 5E

Gene	LFD (fold)		HFD (fold)		HFR (fold)		*P* value (LFD VS. HFD)	*P* value (HFR VS. HFD)
	Mean	SD	Mean	SD	Mean	SD		
Il1b	0.62	0.30	1.22	0.57	0.79	0.23	0.407	0.338
Il6	0.96	0.64	1.07	0.55	0.70	0.09	0.693	0.049
Tnf	0.78	0.41	1.03	0.11	0.63	0.16	0.266	0.012
Ccl2	0.58	0.17	1.11	0.26	0.63	0.17	0.240	0.055
Ifng	0.89	0.33	1.11	0.45	0.69	0.13	0.320	0.260
Ddit3	1.67	0.64	1.58	0.77	0.33	0.08	0.840	0.030
Hspa5	0.96	0.30	1.03	0.30	0.80	0.05	0.752	0.571
Xbp1	1.11	0.19	1.05	0.20	0.71	0.13	0.465	0.055

Supplementary Fig. 1

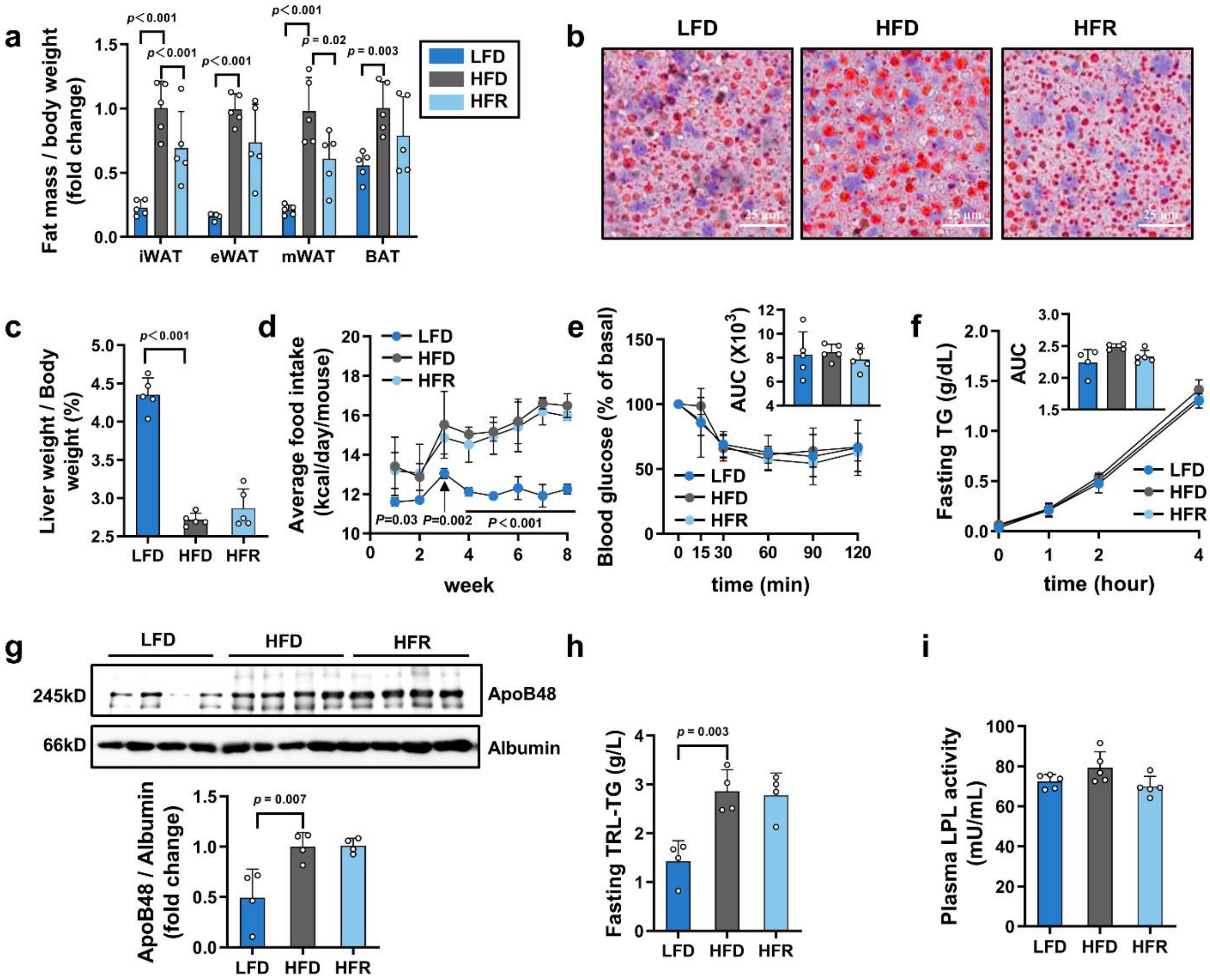

Supplementary Fig. 1 (Related to Fig. 1). REV-I reduces chylomicron production in HFD-challenged mice. (a) The ratio of fat mass to body weight in designated three groups of mice; n=5. **(b)** Representative images of oil-red O staining of liver samples. The scale bar is 25 μm. **(c)** Liver weight to body weight ratio; n=5. **(d)** Food intake of three groups of mice during the entire 8-week period; n=3 for the LFD and HFD groups and n=4 for the HFR group. **(e)** Blood glucose level (% of basal) and AUC during IPITT; n=5. **(f)** Mice fasted overnight were injected intraperitoneally with poloxamer 407 at 1g/kg to block lipolysis of lipoprotein. Plasma was

collected at indicated hours and analyzed for TG concentration; n=4. **(g, h)** Plasma collected 4 h after poloxamer 407 injection was ultra-centrifuged to isolate TRL, and ApoB48 **(g)** and TG concentration **(h)** were measured; n=4. **(i)** LPL activity measured in post-heparin plasma; n=5. iWAT, inguinal white adipose tissue; eWAT, epididymal white adipose tissue; mWAT, mesenteric white adipose tissue; BAT, brown adipose tissue. Statistical significance was evaluated by two-sided one-way ANOVA with Dunnett's *post hoc* test (compared to HFD group). *$P < 0.05$, **$P < 0.01$, ***$P < 0.001$. Data are presented as mean ± SD. Source data are provided as a Source Data file.

Supplementary Fig. 2

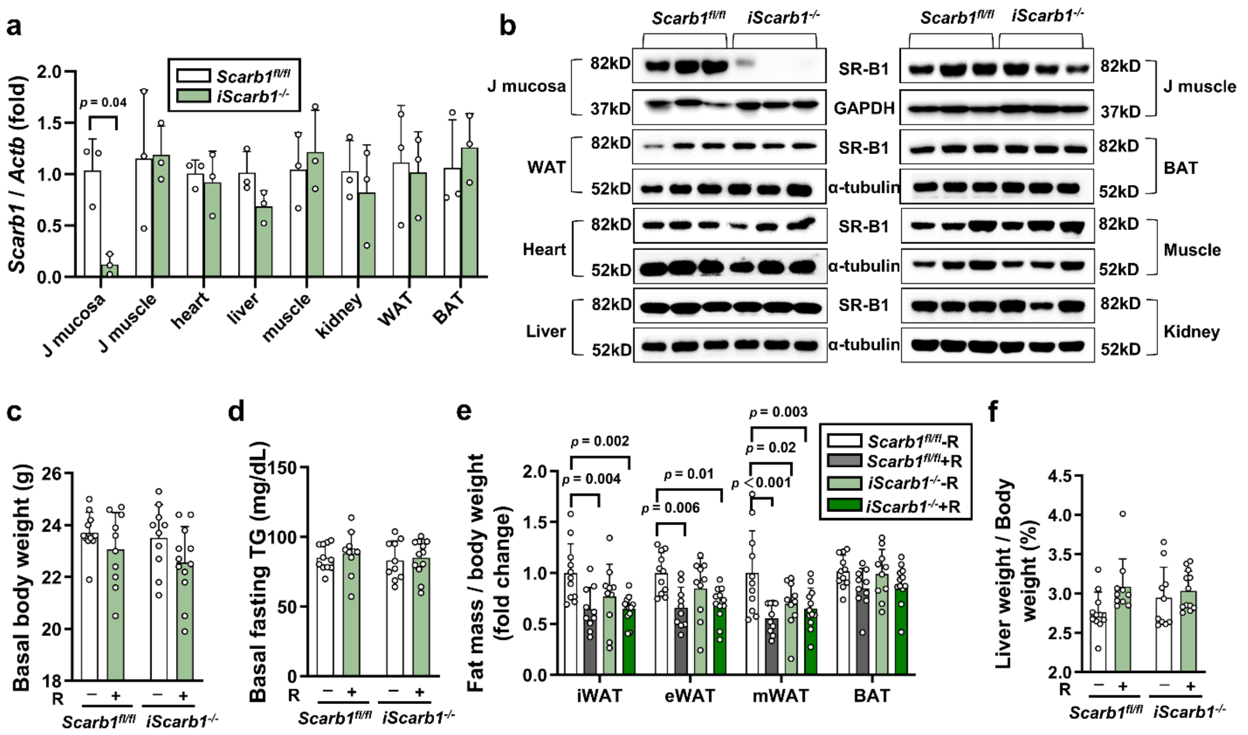

Supplementary Fig. 2 (Related to Fig. 3). Intestinal mucosa-specific SR-B1 KO mice show lack of further response to REV-I. (a) qRT-PCR shows relative mRNA levels of *Scarb1* (normalized by *Actb*, which encodes β-actin) in different tissues of *Scarb1*$^{fl/fl}$ control mice and intestinal SR-B1 knockout (*iScarb1*$^{-/-}$) mice; n=3. **(b)** Detection of SR-B1 in indicated tissues of *Scarb1*$^{fl/fl}$ and *iScarb1*$^{-/-}$ mice by Western blotting; n=3. **(c, d)** Basal body weight **(c)** and fasting TG level **(d)**; n=10-12 as Fig. 3b-c. **(e, f)** Comparison of designated fat mass to body weight ratio **(e)** and liver weight ratio **(f)**; n=10-12 as Fig. 3b-c. J, Jejunum; WAT, white adipose tissue; BAT, brown adipose tissue. Statistical significance was evaluated by two-sided two-way ANOVA with Šidák *post hoc* test. *$P < 0.05$, **$P < 0.01$, ***$P < 0.001$. Data are presented as mean ± SD. Source data are provided as a Source Data file.

Supplementary Fig. 3

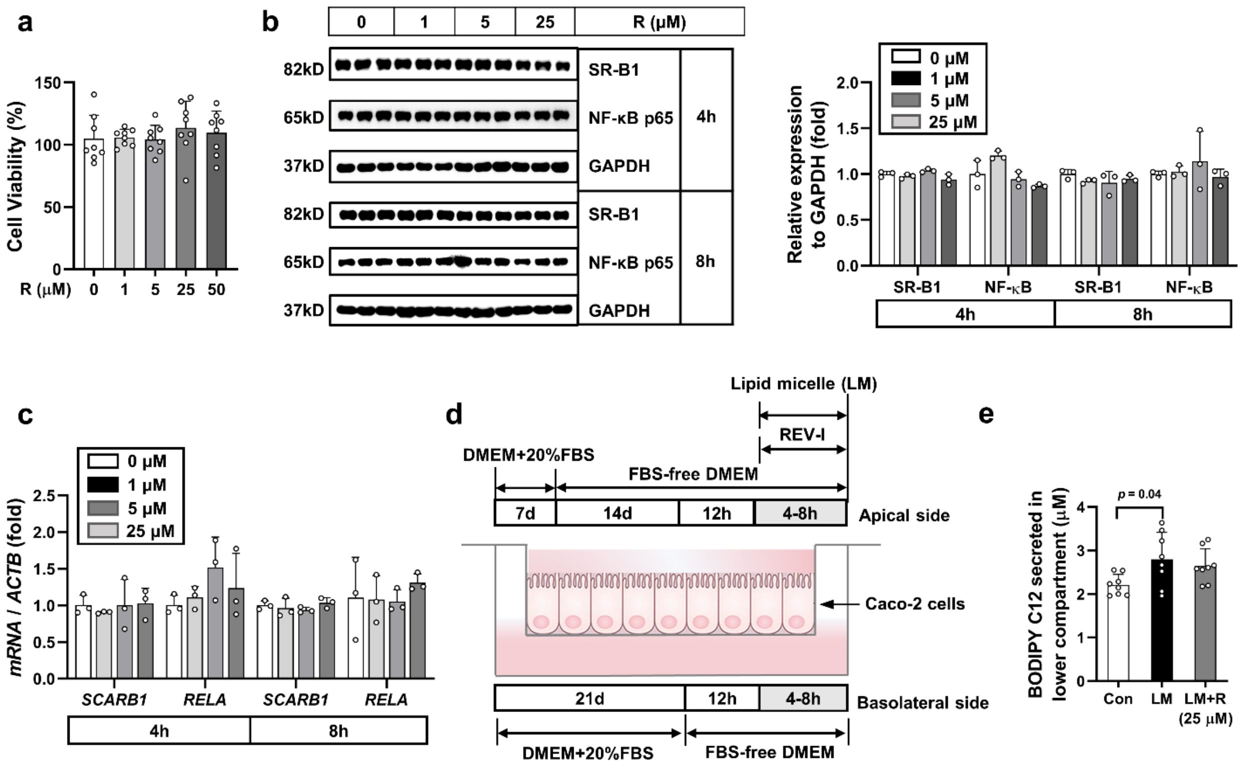

Supplementary Fig. 3. The resveratrol compound does not repress SR-B1 in Caco-2 cells. (a) The effect of different doses of resveratrol on viability of Caco-2 cells detected by MTT; n=8. **(b)** Expression of SR-B1 and NF-κB-p65 in Caco-2 cells treated with resveratrol at 1, 5, or 25 μM for 4 or 8 h; n=3. The whole blot was stripped and re-probed for NF-κB-p65. **(c)** Expression of *SCARB1*, *RELA* (which encodes human NF-κB-p65) genes in Caco-2 cells treated with resveratrol at 1, 5, or 25 μM for 4 or 8 h; n=3. **(d)** Diagram shows the establishment of the intestinal barrier model with differentiated Caco-2 cells. **(e)** Detection of BODIPY labelled C_{12} fatty acid in the lower compartment of the Caco-2 barrier model treated with resveratrol (25 μM) for 8 h; n=8. Statistical significance was evaluated by two-sided one-way ANOVA with Dunnett's *post hoc* test (compared to LM group). *$P < 0.05$. Data are presented as mean ± SD. Source data are provided as a Source Data file.

Supplementary Fig. 4

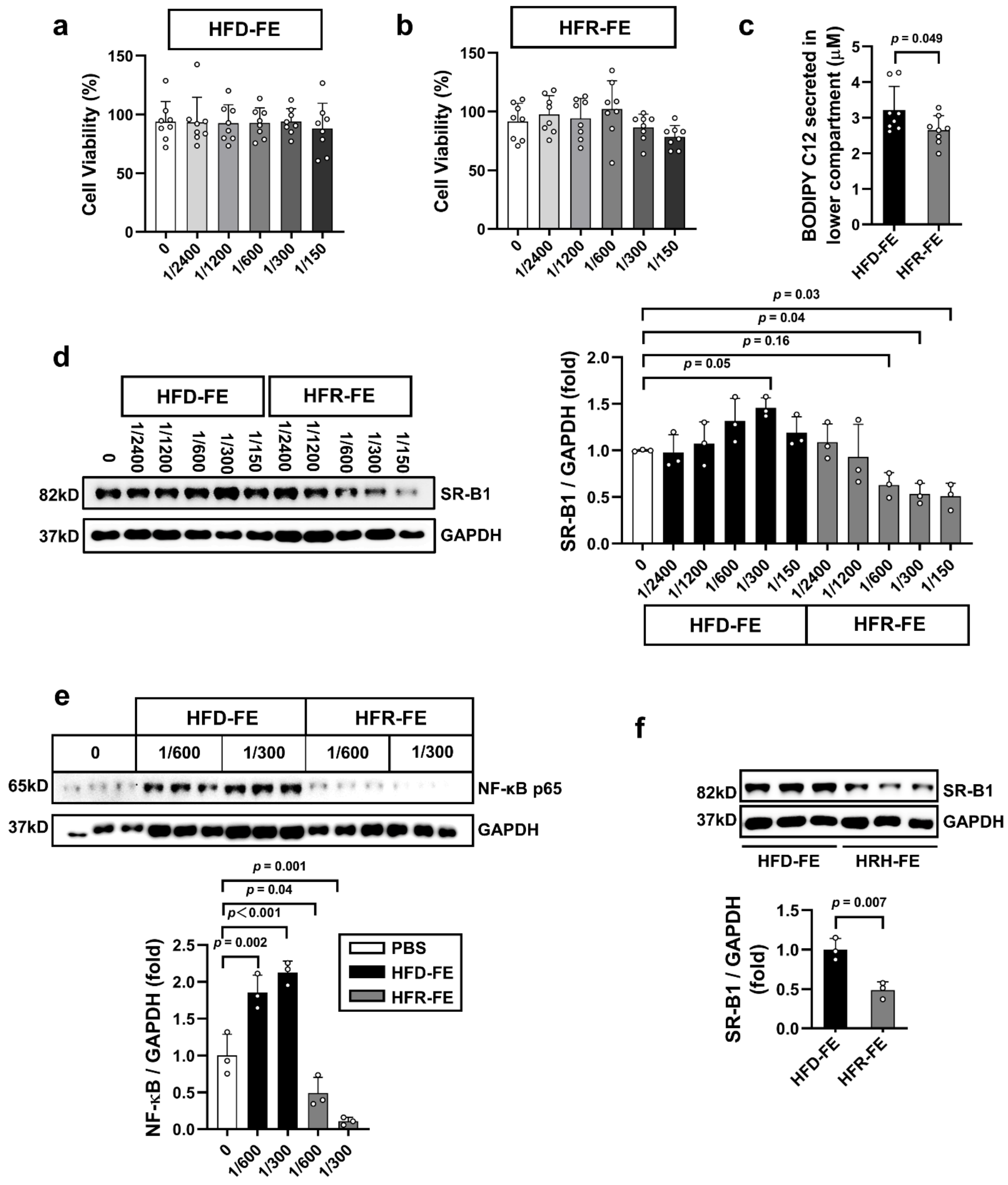

Supplementary Fig. 4. FE represses SR-B1 and chylomicron secretion in Caco-2 cells. (a, b) Effect of indicated FE on Caco-2 cell viability, detected by MTT assay; n=8. **(c)** Detection of BODIPY labelled C_{12} fatty acid in the lower compartment of the Caco-2 barrier model treated with indicated FE (1:1200 dilution) for 4 h. Lipid micelle (LM) was applied to all samples; n=8. **(d)**

Detection of SR-B1 by Western blotting in Caco-2 cells treated with indicated FE with indicated dilution factor for 8 h; n=3. **(e)** Detection of NF-κB p65 by Western blotting in Caco-2 cells treated with indicated FE with indicated dilution factor for 8 h; n=3. **(f)** Detection of SR-B1 in Caco-2 cells treated with heat-treated FE (95℃ for 5 min, diluted at 1:600) for 8 h; n=3. Statistical significance was evaluated by two-sided one-way ANOVA with Dunnett's *post hoc* test (compared to HFD-FE group or PBS group). *$P < 0.05$, **$P < 0.01$, ***$P < 0.001$. Data are presented as mean ± SD. Source data are provided as a Source Data file.

Supplementary Fig. 5

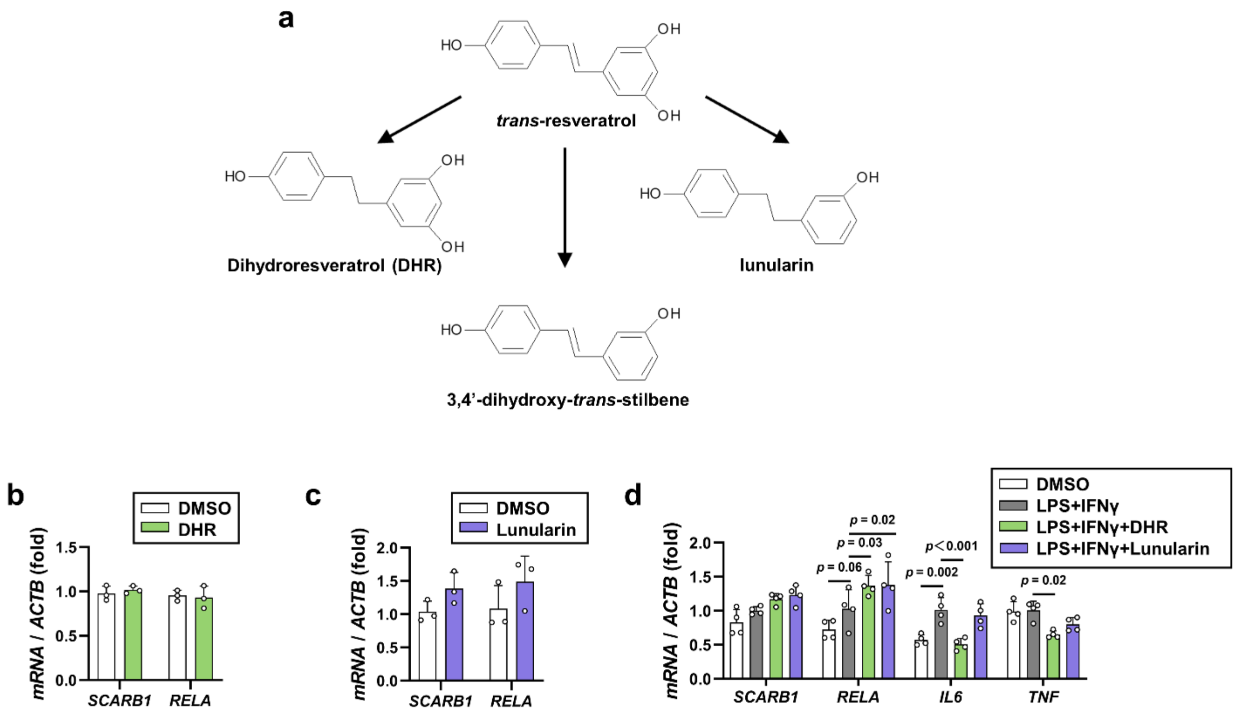

Supplementary Fig. 5. The two commercially available microbial metabolites of resveratrol do not repress SR-B1 in Caco-2 cells. (a) Diagram shows that *trans*-resveratrol can be degraded into three metabolites by gut microbiota, referred to as dihydroresveratrol (DHR), lunularin and 3,4’-dihydroxy-trans-stilbene. The third one is not commercially available. **(b)** The expression of *SCARB1* and *RELA* in Caco-2 cells treated with 25 µM DHR for 8 h; n=3. **(c)** The expression of *SCARB1* and *RELA* in Caco-2 cells treated with 25 µM lunularin for 8h; n=3. **(d)** The expression of *SCARB1*, *RELA*, *IL6* (which encodes human IL-6) and *TNF* (which encodes human TNF-α) in Caco-2 cells treated with 25 µM DHR or 25 µM lunularin in the presence of LPS (200ng/mL) and IFNγ (200ng/mL) treatment for 8 h; n=4. Statistical significance was evaluated by two-sided one-way ANOVA with Dunnett's *post hoc* test (compared to LPS+IFNγ group). *$P < 0.05$, **$P < 0.01$, ***$P < 0.001$. Data are presented as mean ± SD. Source data are provided as a Source Data file.

Supplementary Fig. 6

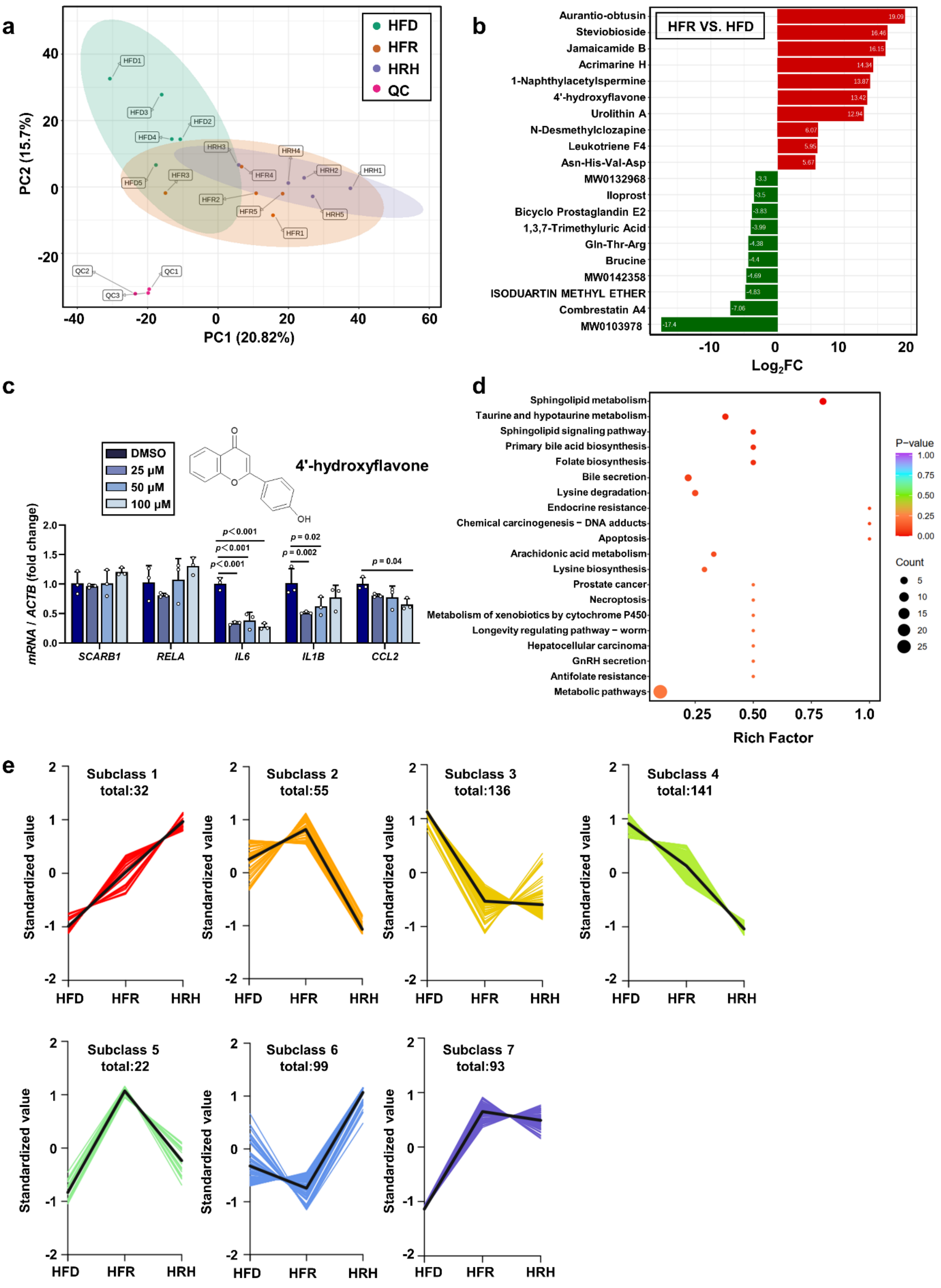

Supplementary Fig. 6 (Related to Fig. 7). REV-I attenuates HFD-induced fecal CDCA elevation. (a) Principal component analysis (PCA) plot. Sterile FEs from the designated group were subjected to untargeted metabolomics analysis. HFR, HFD fed mice with REV-I. HRH, sterile FE isolated from mice on HFR treatment was heated at 95°C for 5 min; n=5. **(b)** The top 10 increased and decreased metabolites (as indicated by Log2FC) in fecal extract from mice of HFR group compared to mice of HFD group; n=5. **(c)** The expression of *SCARB1*, *RELA*, *IL6*, *IL1B* (which encodes human IL-1β) and *CCL2* (which encodes human MCP-1) in Caco-2 cells treated with 4'-hydroxyflavone (of which the molecular structure was shown on the top) at 25, 50 or 100 μM for 8 h; n=3. **(d)** KEGG pathway enrichment analysis shows the top 20 regulated metabolic pathways by REV-I. Rich Factor was determined by the ratio of differential metabolites detected vs total metabolites in that pathway. **(e)** K-Means cluster analysis divided all the differential metabolites into 7 subclasses according to their relative abundance in different groups. Only subclasses 3 and 7 show comparable levels in HFR and HRH groups. Statistical significance was evaluated by two-sided one-way ANOVA with Dunnett's *post hoc* test (compared to control group for Panel c). *$P < 0.05$, **$P < 0.01$, ***$P < 0.001$. Data are presented as mean ± SD. Source data are provided as a Source Data file.

Supplementary Fig. 7

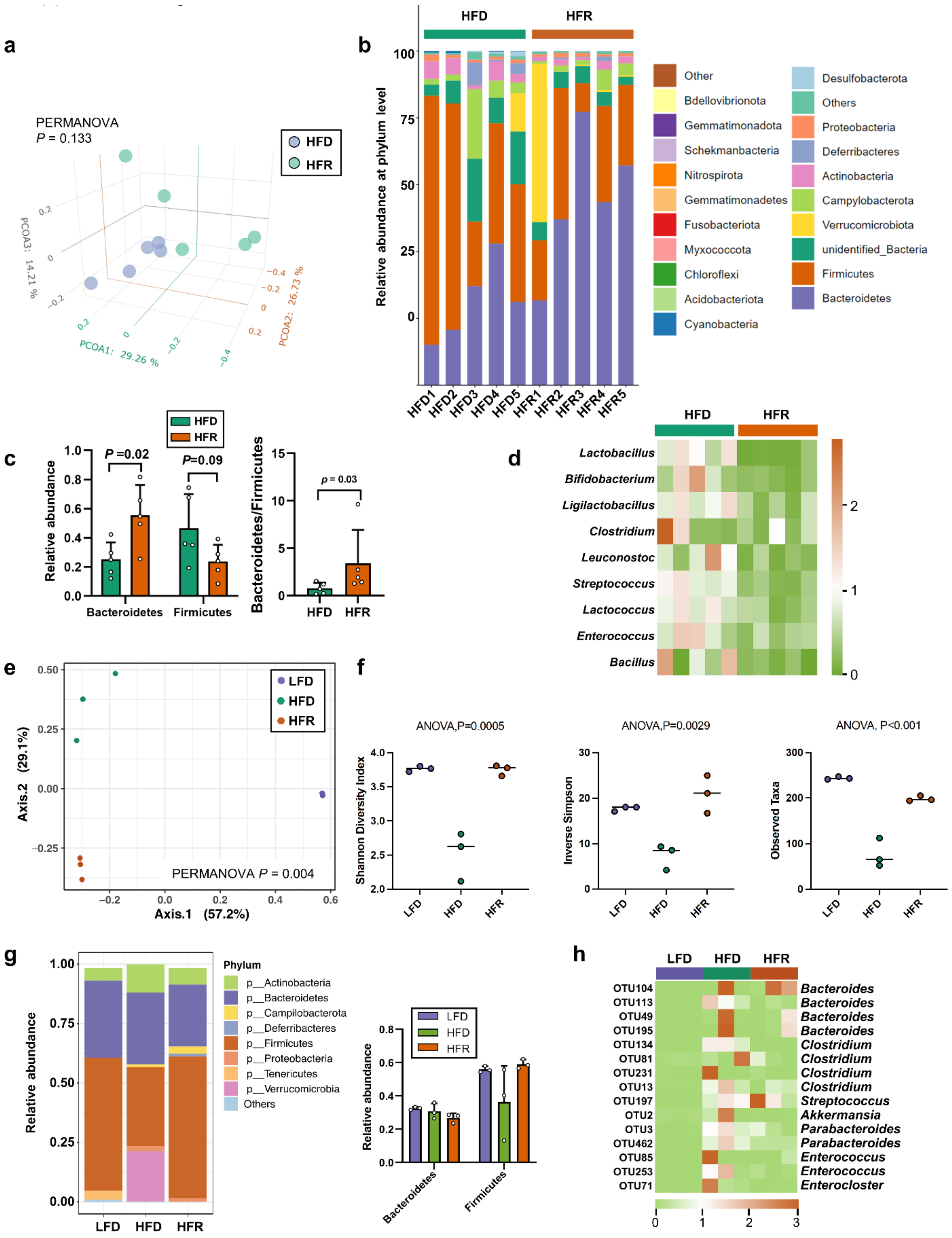

Supplementary Fig. 7. REV-I "re-shapes" gut microbiome composition. Panels a-d for feces obtained from mice on HFD or on HFD with REV-I (designated as HFR). Panels e-h for mouse caecum contents from designated group of mice. (a) Bray-Curtis beta-diversity visualized with three-dimensional PCoA by PERMANOVA test; n=5. **(b)** The stacking histogram showing taxonomic summary of phyla composition in feces from HFD and HFR groups; n=5. **(c)** The relative abundance of fecal *Bacteroidetes* and *Firmicutes* (left panel) and *Bacteroidetes* to *Firmicutes* ratio (right panel); n=5. Two-sided student-t test and Mann-Whitney nonparametric test were conducted for comparisons. **(d)** The heatmap shows the relative abundance of BSH-producing genera; n=5. **(e)** Bray-Curtis beta-diversity visualized with PCoA; n=3. **(f)** Alpha diversity differences were assessed by Shannon diversity index, Inverse Simpson and Observed richness metrics, respectively; n=3. **(g)** Taxonomic summary of phyla composition in the caecum content, as well as relative abundance of *Bacteroidetes* and *Firmicutes*; n=3. **(h)** The heatmap shows the relative abundance of BSH-producing genera; n=3. *$P < 0.05$. Data are presented as mean ± SD.
