# Supplementary material for: Resveratrol intervention attenuates chylomicron secretion via repressing intestinal FXR-induced expression of scavenger receptor SR-B1
Source: Nat Commun. 2023 May 9;14:2656. doi: 10.1038/s41467-023-38259-1 (PMC10169763; doi:10.1038/s41467-023-38259-1)
Supplement: Supplementary file 3 — Description of Additional Supplementary Files [file 41467_2023_38259_MOESM3_ESM.docx]

**Description of Additional Supplementary Files**

File Name: Supplementary Data 1

Description: all metabolites detected in the untargeted metabolomics profiling

File Name: Supplementary Data 2

Description: differential metabolites between HFD and HFR groups

File Name: Supplementary Data 3

Description: differential metabolites between HFD and HRH groups

File Name: Supplementary Data 4

Description: 30 key differential metabolites with their retention time provided by the service company
