# Supplementary material for: Resveratrol intervention attenuates chylomicron secretion via repressing intestinal FXR-induced expression of scavenger receptor SR-B1
Source: Nat Commun. 2023 May 9;14:2656. doi: 10.1038/s41467-023-38259-1 (PMC10169763; doi:10.1038/s41467-023-38259-1)
Supplement: Supplementary file 8 — Reporting Summary [file 41467_2023_38259_MOESM8_ESM.pdf]

nature portfolio

Corresponding author(s): Khosrow Adeli, Wenhua Ling, Tianru Jin

Last updated by author(s): Apr 18, 2023

Reporting Summary

Nature Portfolio wishes to improve the reproducibility of the work that we publish. This form provides structure for consistency and transparency in reporting. For further information on Nature Portfolio policies, see our Editorial Policies and the Editorial Policy Checklist.

Statistics

For all statistical analyses, confirm that the following items are present in the figure legend, table legend, main text, or Methods section.

n/a	Confirmed	
☐	☒	The exact sample size (*n*) for each experimental group/condition, given as a discrete number and unit of measurement
☐	☒	A statement on whether measurements were taken from distinct samples or whether the same sample was measured repeatedly
☐	☒	The statistical test(s) used AND whether they are one- or two-sided<br>*Only common tests should be described solely by name; describe more complex techniques in the Methods section.*
☒	☐	A description of all covariates tested
☐	☒	A description of any assumptions or corrections, such as tests of normality and adjustment for multiple comparisons
☐	☒	A full description of the statistical parameters including central tendency (e.g. means) or other basic estimates (e.g. regression coefficient) AND variation (e.g. standard deviation) or associated estimates of uncertainty (e.g. confidence intervals)
☐	☒	For null hypothesis testing, the test statistic (e.g. *F*, *t*, *r*) with confidence intervals, effect sizes, degrees of freedom and *P* value noted<br>*Give P values as exact values whenever suitable.*
☒	☐	For Bayesian analysis, information on the choice of priors and Markov chain Monte Carlo settings
☐	☒	For hierarchical and complex designs, identification of the appropriate level for tests and full reporting of outcomes
☒	☐	Estimates of effect sizes (e.g. Cohen's *d*, Pearson's *r*), indicating how they were calculated

Our web collection on statistics for biologists contains articles on many of the points above.

Software and code

Policy information about availability of computer code

Data collection	In metabolomics profiling, Cutadapt v.2.6 was used to trim the last base from all reads. QIIME1 was used to align OTUs.<br>Analyst 1.6.3 software (SCIEX) was used for the widely targeted metabolomics profiling and bile acid data acquisition.<br>Multiquant 3.0.3 software (Sciex) was used to quantify all metabolites.
Data analysis	Image J software (version 1.8.0, https://imagej.nih.gov/ij/) was used to semi-quantify gray values of western blots. Statistical analyses were performed with GraphPad Prism 8.0 (GraphPad Software, La Jolla, CA, USA) or with R (version 3.5.1, https://www.r-project.org/)

For manuscripts utilizing custom algorithms or software that are central to the research but not yet described in published literature, software must be made available to editors and reviewers. We strongly encourage code deposition in a community repository (e.g. GitHub). See the Nature Portfolio guidelines for submitting code & software for further information.

Data

Policy information about availability of data

All manuscripts must include a data availability statement. This statement should provide the following information, where applicable:

- Accession codes, unique identifiers, or web links for publicly available datasets
- A description of any restrictions on data availability
- For clinical datasets or third party data, please ensure that the statement adheres to our policy

SINTAX was used to assign taxonomy using USEARCH and the Ribosomal Database Project database v18 available through UNOISE (http://rdp.cme.msu.edu/).

The KEGG (Kyoto Encyclopedia of Genes and Genomes) database (http://www.genome.Jp/kegg/) was used to find enriched metabolic signaling pathways.
PROMO (version 8.3, http://alggen.lsi.upc.es/) was used to analyze binding motifs of transcriptional activators.
Source data and uncropped blots are provided with this paper as supplementary files named as Data_S1_Source_Data.zip.
The 16S rRNA sequencing raw data have been deposited in Sequence Read Archive (SRA) with the accession number PRJNA859433.
The untargeted metabolomics profiling processed data have been deposited in Figshare with the DOI number 10.6084/m9.figshare.20325402.v1 and the raw data have been deposited in MetaboLights with the accession number MTBLS7654.

Human research participants

Policy information about studies involving human research participants and Sex and Gender in Research.

Reporting on sex and gender	The study did not involve human research participants
Population characteristics	not applicable
Recruitment	not applicable
Ethics oversight	not applicable

Note that full information on the approval of the study protocol must also be provided in the manuscript.

Field-specific reporting

Please select the one below that is the best fit for your research. If you are not sure, read the appropriate sections before making your selection.

☒ Life sciences ☐ Behavioural & social sciences ☐ Ecological, evolutionary & environmental sciences

For a reference copy of the document with all sections, see nature.com/documents/nr-reporting-summary-flat.pdf

Life sciences study design

All studies must disclose on these points even when the disclosure is negative.

Sample size	No sample-size calculation was performed. Based on community standards, n=6 or more mice per group was acceptable for animal studies (Nat Commun. 2019;10(1):4971)
Data exclusions	The values which are more than mean±3SD are regarded as outliers in statistics and were excluded. The criteria is pre-established.
Replication	Generally, each experiment was repeated for three times expect for mice experiments and all attempts at replication were successful with similar results.
Randomization	All samples were randomly allocated among the groups before experiments.
Blinding	Complete blinding is not possible in animal study and cell culture due to different treatment to different groups. We tried to number the mice randomly when collecting tissues and in this way, investigators are blind to the group of one specific sample and can avoid some biases.

Reporting for specific materials, systems and methods

We require information from authors about some types of materials, experimental systems and methods used in many studies. Here, indicate whether each material, system or method listed is relevant to your study. If you are not sure if a list item applies to your research, read the appropriate section before selecting a response.

Materials & experimental systems

n/a	Involved in the study	
☐	☒	Antibodies
☐	☒	Eukaryotic cell lines
☒	☐	Palaeontology and archaeology
☐	☒	Animals and other organisms
☒	☐	Clinical data
☒	☐	Dual use research of concern

Methods

n/a	Involved in the study	
☒	☐	ChIP-seq
☒	☐	Flow cytometry
☒	☐	MRI-based neuroimaging

Antibodies

Antibodies used	Rabbit anti-SR-B1 (Abcam, Cat#ab217318, 1:3000); Goat anti-ApoB (Midland Bioproducts, Cat#MBC-APB-G1, 1:1000); Goat anti-alpha-albumin (Nittobo America, Cat#MBC-ALB-G1, 1:1000); Rabbit anti-NF-kappaB-p65 (Cell Signaling Technology, Cat#8242, 1:1000); Rabbit anti-GAPDH (Cell Signaling Technology, Cat#2118, 1:1000); Mouse anti-beta-actin (Cell Signaling Technology, Cat#3700, 1:1000); Rabbit anti-alpha-tubulin (Cell Signaling Technology, Cat#2144, 1:1000); Alexa Fluor 555 conjugated goat anti-rabbit IgG (ThermoFisher Scientific, Cat#A-21428, 1:200); ImmPress Horse anti-rabbit IgG (Novus Biologicals, Cat#MP-7401-NB, 1:200); horse anti-mouse IgG-HRP (Cell Signaling Technology, Cat#7076, 1:2000); goat anti-rabbit IgG-HRP (Cell Signaling Technology, Cat#7074, 1:2000); donkey anti-goat IgG-HRP (Santa Cruz Biotechnology, Cat#sc-2020, 1:2000)
Validation	Antibodies used were commercially available and were validated in multiple previous studies.<br>Rabbit anti-SR-B1 reacts with mouse, rat and human species, and the applications include WB, IHC, ICC/IF and IP;<br>Goat anti-ApoB and anti-alpha-albumin reacts with human species but also reacts with mouse species by validation in the current study, and the antibodies were used for WB;<br>Rabbit anti-NF-kappaB-p65 reacts with human, mouse, rat, hamster, monkey and dog species, and the applications include WB, IHC, ICC/IF, IP and Flow Cytometry (FC);<br>Rabbit anti-GAPDH reacts with human, mouse, rat, monkey, bovine and pig, and the applications include WB, IHC, ICC/IF and FC;<br>Mouse anti-beta-actin reacts with human, mouse, rat, hamster, monkey and dog, and the applications include WB, IHC, ICC/IF and FC; Rabbit anti-alpha-tubulin reacts with human, mouse, rat, monkey, D. melanogaster and bovine, and the applications include WB, IHC, ICC/IF and FC

Eukaryotic cell lines

Policy information about cell lines and Sex and Gender in Research

Cell line source(s)	Human Caco-2 cells (ATCC, Cat#HTB-37, isolated from colon tissue of a 72-year-old white male person)
Authentication	The cell line used was authenticated by STR profiling
Mycoplasma contamination	The cell line was tested negative for mycoplasma contamination.
Commonly misidentified lines (See ICLAC register)	No misidentified line was used in the study

Animals and other research organisms

Policy information about studies involving animals; ARRIVE guidelines recommended for reporting animal research, and Sex and Gender in Research

Laboratory animals	6-week-old male C57BL/6J mice; 6-week-old intestinal mucosa-specific SR-B1 knock-out male mice on C57BL/6J background which were generated by mating SR-B1 floxed mice provided by Dr. Philip W. Shaul and Villin-Cre mice (Jackson Laboratory, Stock#021504). Mice were group-housed in individually ventilated cages under controlled temperature and humidity with a 12-h light–dark cycle.
Wild animals	The study did not involve wild animals.
Reporting on sex	Only male C57BL/6J mice were used because female mice are not susceptible to diet-induced obesity and metabolic disorders, such as insulin resistance and hypertriglyceridemia.
Field-collected samples	The study did not involve samples collected from the field.
Ethics oversight	All animal protocols were approved by the University Health Network Animal Care Committee, or The Hospital for Sick Children Animal Care Committee, or the Animal Ethics Committee of Sun Yat-sen University.

Note that full information on the approval of the study protocol must also be provided in the manuscript.
