# Supplementary material for: Resveratrol intervention attenuates chylomicron secretion via repressing intestinal FXR-induced expression of scavenger receptor SR-B1
Source: Nat Commun. 2023 May 9;14:2656. doi: 10.1038/s41467-023-38259-1 (PMC10169763; doi:10.1038/s41467-023-38259-1)

Figure 1l

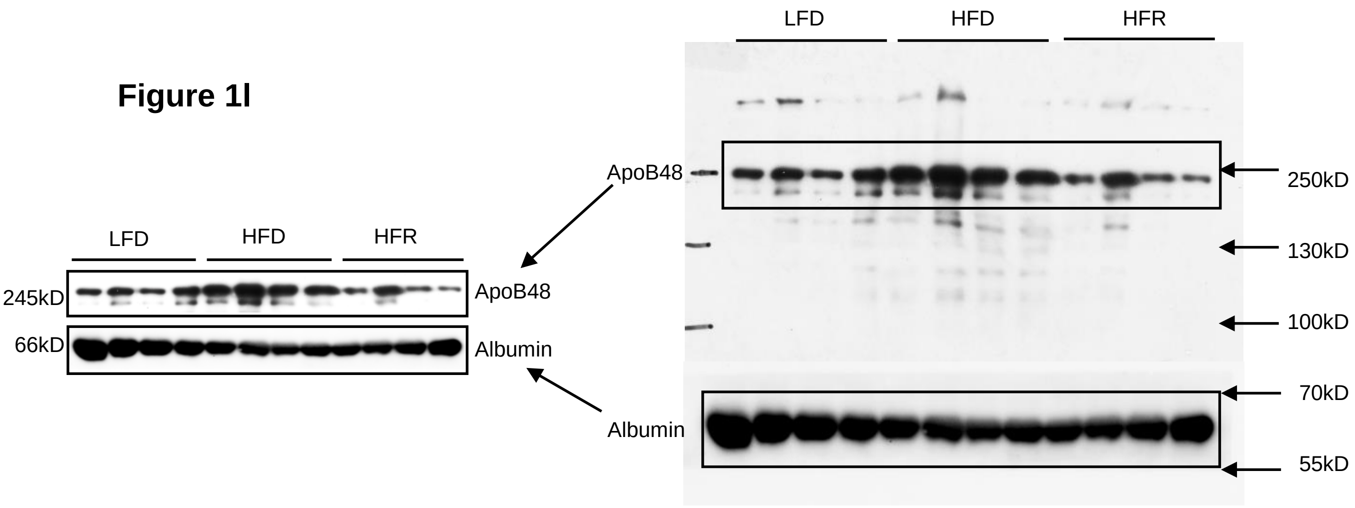

Figure 2f

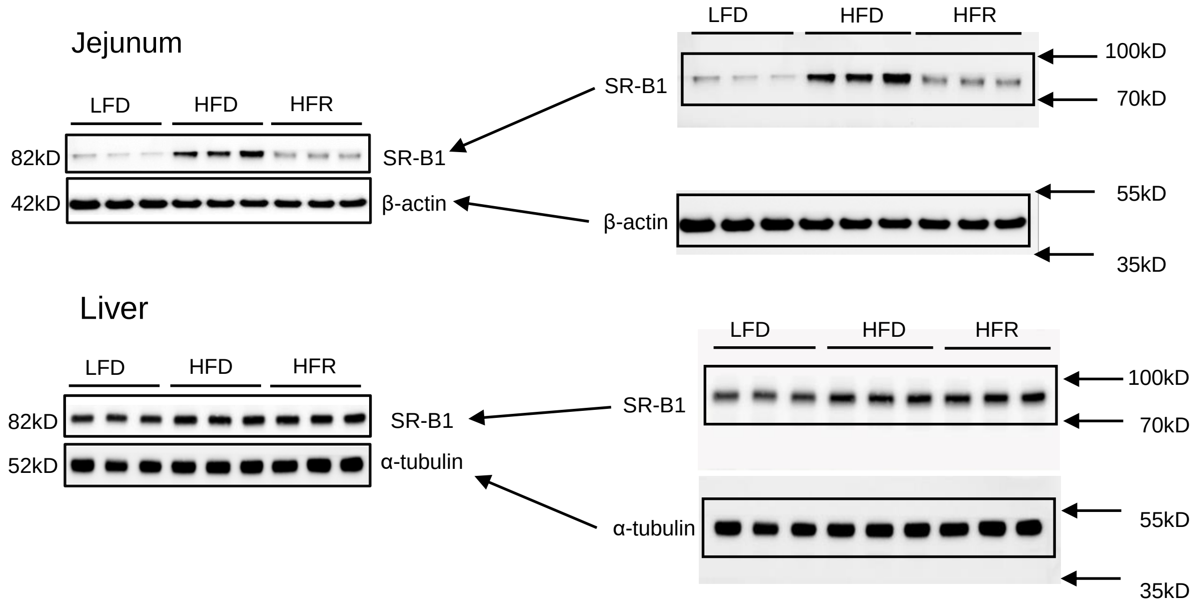

Figure 3j

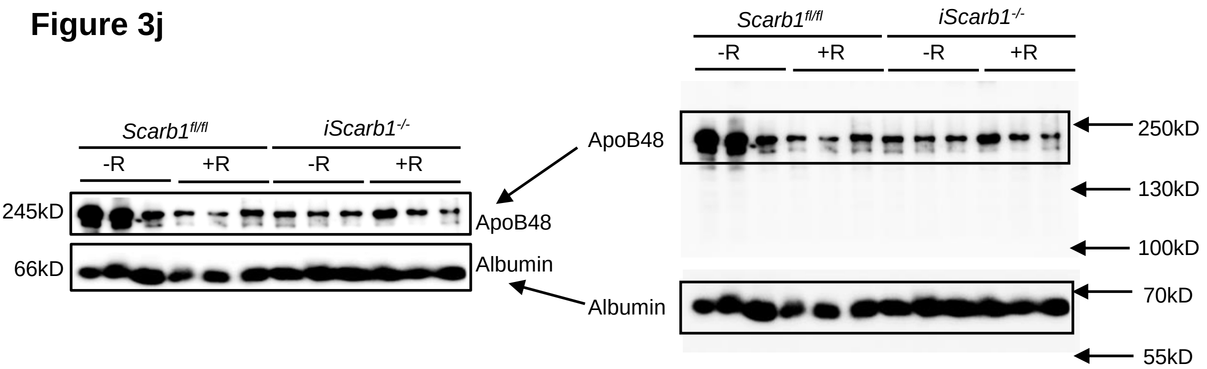

Figure 4j

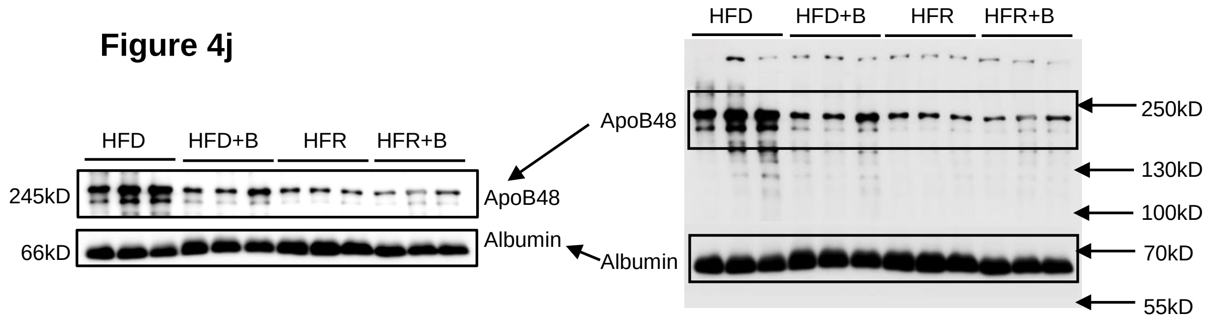

Figure 5a

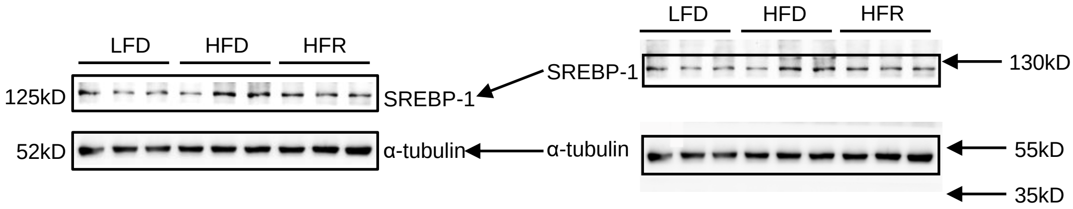

Figure 5d

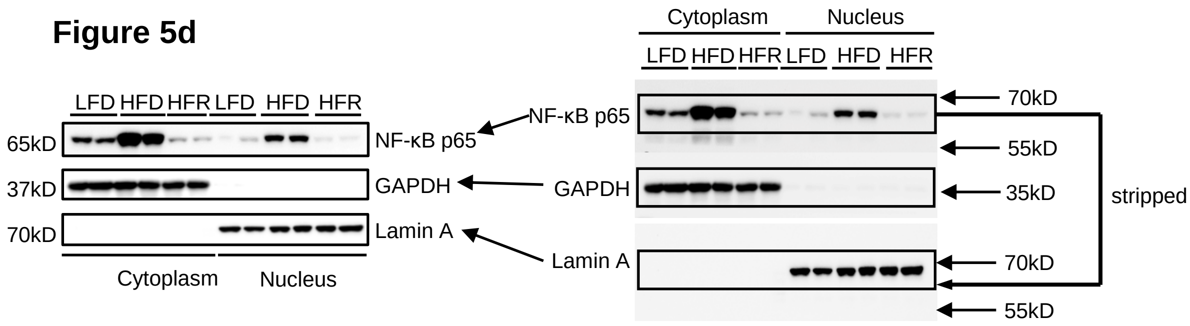

Figure 5f

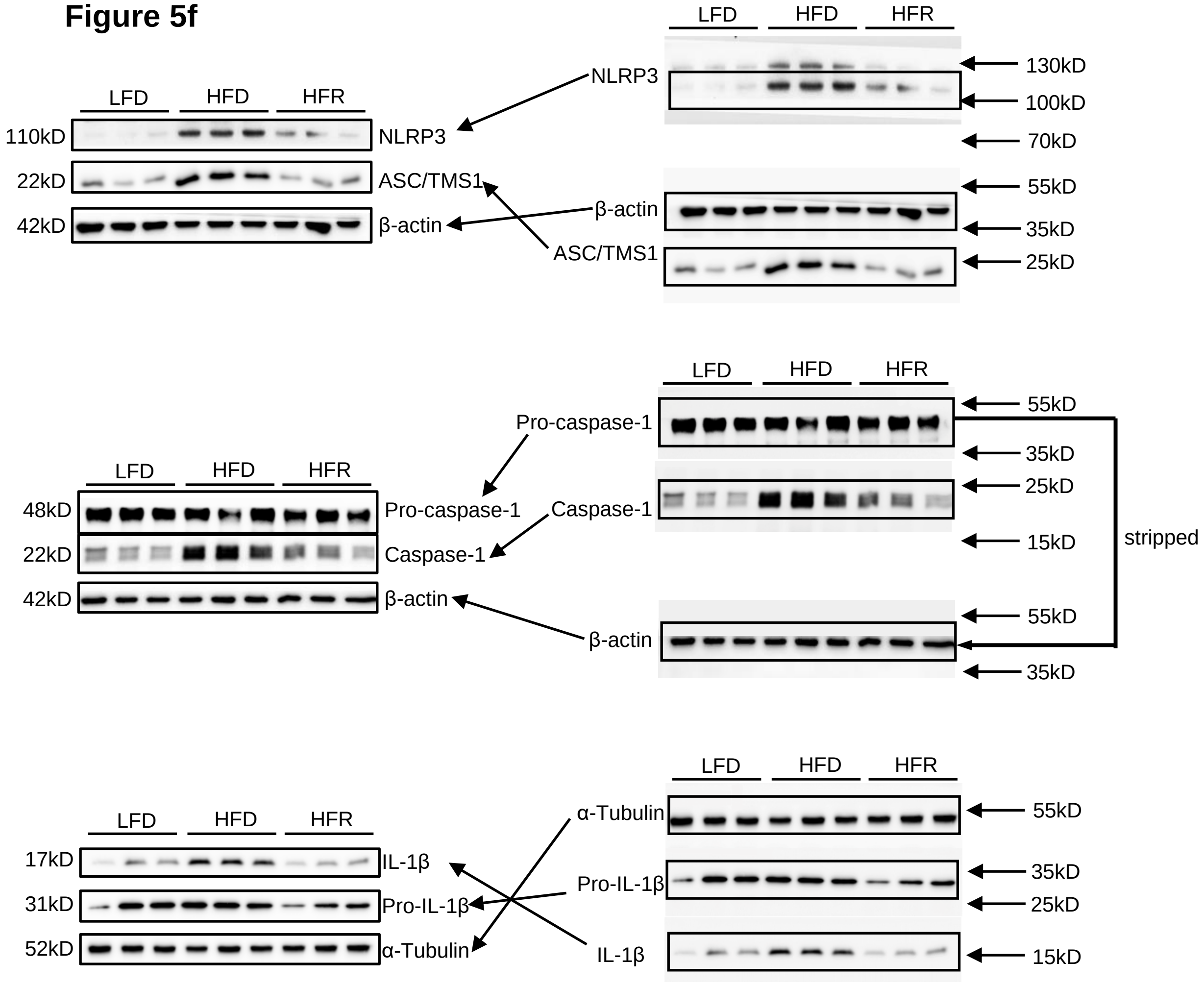

LFD
HFD
HFR
110kD
NLRP3
22kD
ASC/TMS1
42kD
β-actin
NLRP3
β-actin
ASC/TMS1
130kD
100kD
70kD
55kD
35kD
25kD
48kD
Pro-caspase-1
22kD
Caspase-1
42kD
β-actin
Pro-caspase-1
Caspase-1
β-actin
55kD
35kD
25kD
15kD
55kD
35kD
stripped
17kD
IL-1β
31kD
Pro-IL-1β
52kD
α-Tubulin
α-Tubulin
Pro-IL-1β
IL-1β
55kD
35kD
25kD
15kD

Figure 5g

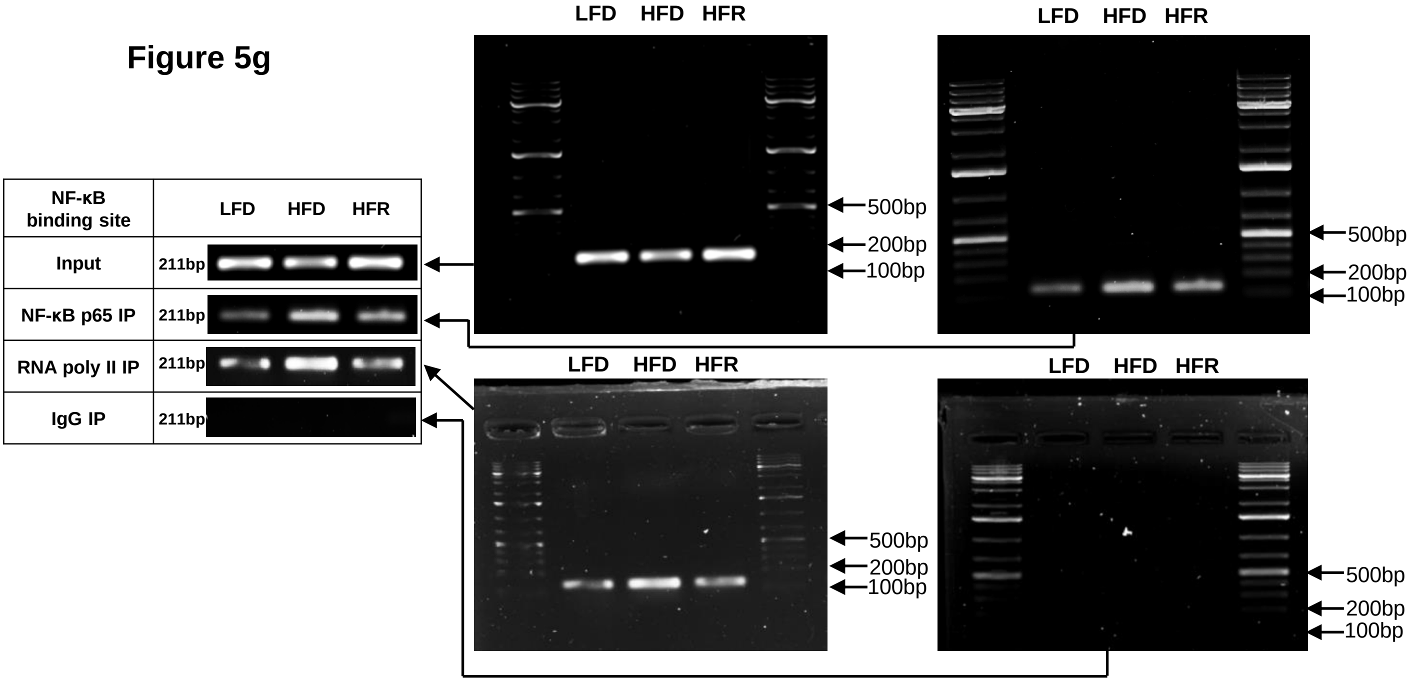

Figure 5h

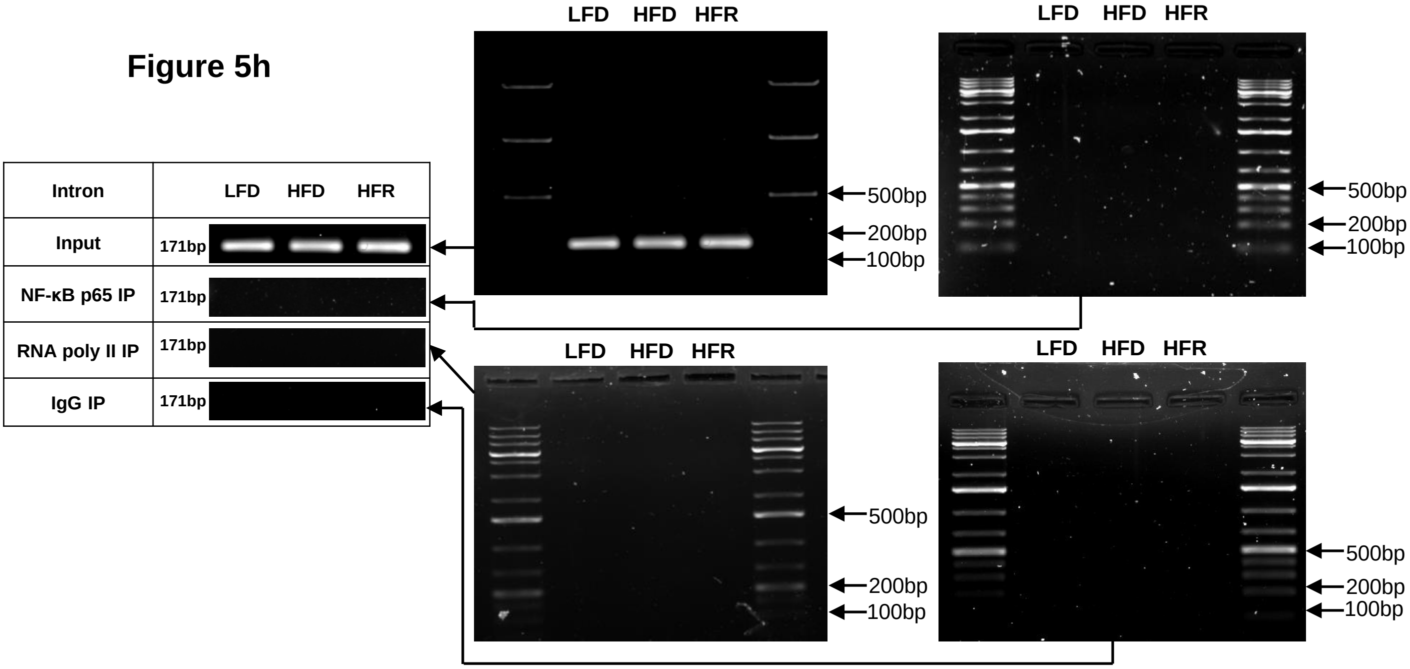

Figure 6h

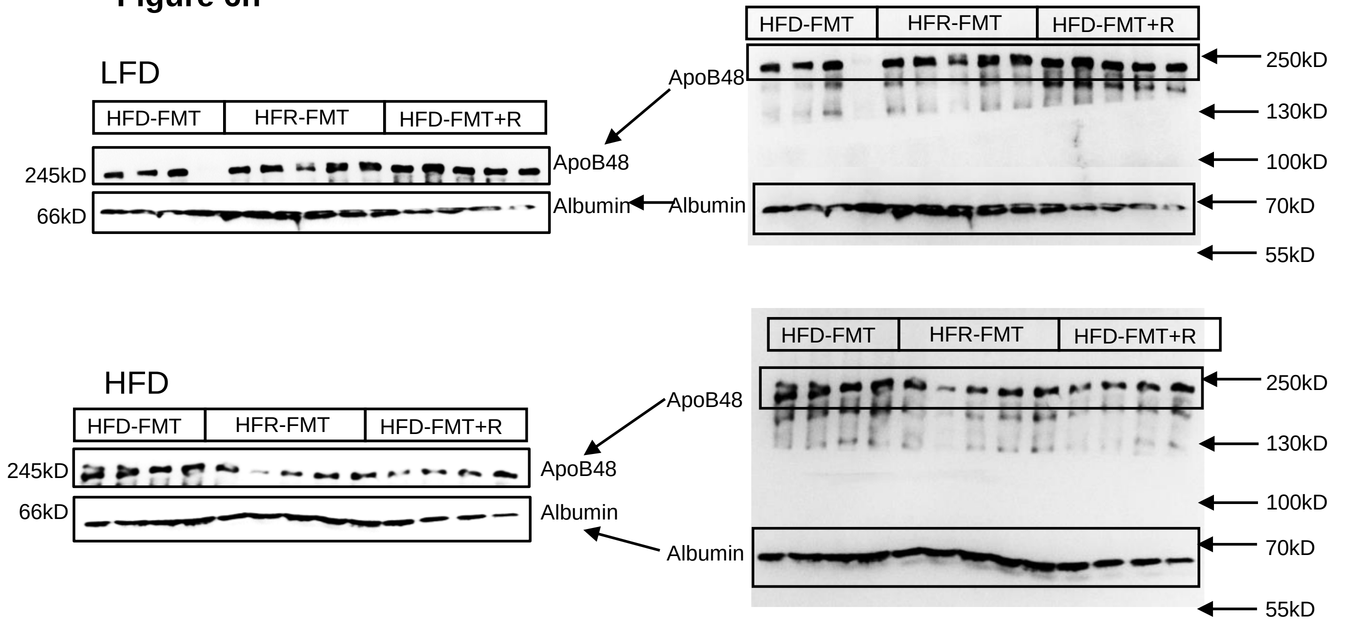

Figure 6i

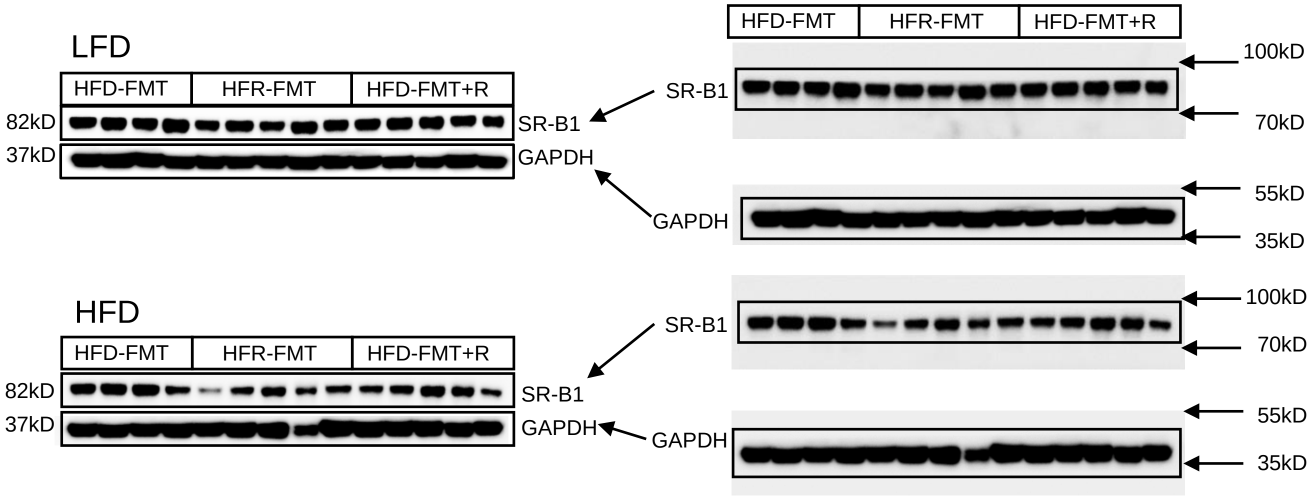

Figure 8l

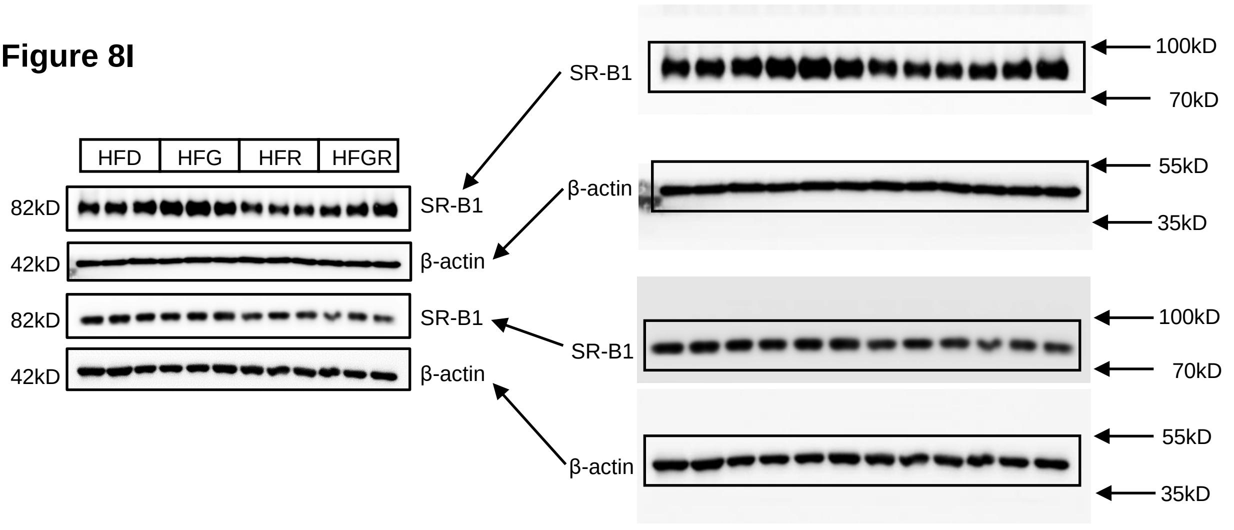

Supplement: Supplementary file 9 — Source Data [file 41467_2023_38259_MOESM9_ESM.zip › uncropped blots.pdf]
